# Association between uncertainty regarding malignant risk and anxiety in patients with pulmonary nodules: a serial mediation analysis of illness perception and rumination

**DOI:** 10.3389/fpsyt.2026.1957701

**Published:** 2026-10-15

**Authors:** Shanshan Wu, Min Liu, Song Yi, Rui Wang, Zhiqiang Song, Feng Xie, Wei Chen, Hong Qu, Yurong Huang

**Affiliations:** 1 Department of Oncology, Xinjiang Production and Construction Corps Hospital (The Second Affiliated Hospital of Shihezi University Medical College), Urumqi, China; 2 Department of Respiratory and Critical Care Medicine, Xinjiang Production and Construction Corps Hospital, Urumqi, China

**Keywords:** anxiety, chain mediation, disease perception, pulmonary nodules, ruminating thinking, uncertainty of malignant risk

## Abstract

**Objective:**

This study examined the relationship between uncertainty regarding the risk of malignancy after pulmonary nodule detection and anxiety, and the serial indirect effects of illness perception and ruminative thinking.

**Methods:**

A total of 308 patients with pulmonary nodules who were admitted to our hospital between January 2023 and January 2026 were included in the study. General information was collected, and participants were assessed using the MUIS, BBIPQ, RRS, and the Chinese version of the GAD-7. Correlation and serial indirect effect analyses were performed using SPSS 26.0 and the PROCESS macro (Model 6). Bootstrap resampling with 5,000 samples was used to assess the stability of indirect effects.

**Results:**

Among the 308 patients with pulmonary nodules, the MUIS scores ranged from 24 to 86, with a mean of 56.10 ± 12.21. The BIPQ scores ranged 3 to76, with a mean of 43.26 ± 10.67, the RRS scores ranged from 22 to 72, with a mean of 44.66 ± 10.60, and the GAD-7 scores ranged from 1 to16, with a mean of 8.84 ± 2.62. No significant differences in GAD-7 scores were observed among patients with different demographic or disease characteristics (*P*>0.05). Pearson’s correlation analysis showed that MUIS scores were positively correlated with BIPQ, RRS and GAD-7 scores (*r* = 0.687, 0.577, and 0.745, *P* < 0.05). BIPQ score was positively correlated with RRS and GAD-7 score (*r* = 0.577; 0.601, *P* < 0.05). RRS score was positively correlated with GAD-7 score (*r* = 0.574, *P* < 0.05). The MUIS had a significant direct effect on GAD-7, accounting for 75.63% of the total effect. The indirect effect consists of three paths: ① MUIS → RRS → GAD-7, with an indirect effect of 0.014, accounting for 8.75% of the total effect; ② MUIS → BIPQ → GAD-7, with an indirect effect of 0.016, accounting for 10.00% of the total effect; and ③ MUIS → BIPQ → RRS → GAD-7, with an indirect effect of 0.009, accounting for 5.62% of the total effect.

**Conclusion:**

After pulmonary nodule detection, uncertainty regarding the risk of malignancy is positively associated with anxiety, partly explained by the serial indirect effects of illness perception and ruminative thinking, consistent with the hypothesized model.

With the wide application of low-dose computed tomography (CT) in lung cancer screening and improvements in public health awareness, the detection rate of pulmonary nodules is increasing annually. A study published in 2024 reported that the global prevalence rate of pulmonary nodules is appropximately 27%, and the prevalence rate in men is slightly higher than that in women (1). An epidemiological survey in China showed that the prevalence rate of pulmonary nodules is appropximately 55.9% (2). However, because imaging features cannot completely rule out the possibility of malignancy in a short period of time, most patients with pulmonary nodules require follow-up and further differential diagnosis. At this stage, the nature of the nodules is unknown, and patients are in an uncertain state of malignant risk (3). Uncertainty regarding malignant risk refers to a cognitive state in which individuals cannot accurately predict the development process, treatment effect, and outcome of the disease (4). It is pointed out that the uncertainty regarding malignant risk is one of the main psychological stressors of people with positive cancer screening, which is closely related to negative emotions such as anxiety and depression (5). However, the internal relationship between the uncertainty regarding the risk of malignancy and anxiety in patients with pulmonary nodules is unclear, and it is of great significance to explore the internal relationship between them to formulate targeted psychological intervention measures. Illness perception is the core content of self-regulation theory, referring to an individual’s cognition, understanding, and evaluation of the disease they are suffering from. This theory holds that when individuals face disease threats, they form cognitive representations of the disease based on their knowledge, experience, and information. These cognitive representations may influence individuals’ emotional responses and coping behaviors (6). Jaltuszewska et al. (7) confirmed that an individual’s perception of negative diseases increases the risk of negative emotions, such as anxiety and depression. Ruminative thinking is a cognitive style in which individuals repeatedly pay attention to their negative emotions and their causes and consequences, which prolongs and aggravates their negative emotions and is an important risk factor for the occurrence and development of anxiety (8).

Most existing studies have separately examined the pairwise associations among illness uncertainty, illness perception, ruminative thinking, and anxiety. However, the sequential relationships among these four factors have not been systematically investigated (9, 10). Self-regulation theory suggests that when individuals face a health threat, they first develop cognitive representations of the illness, referred to as illness perceptions, and subsequently select and adjust their cognitive and coping strategies based on these representations. Ruminative thinking, a cognitive-emotional coping strategy characterized by a repetitive focus on negative content and its causes and consequences, is typically activated following the formation of illness perceptions. Thus, theoretically, illness perception precedes ruminative thinking (6, 8). Several plausible sequences among these variables may nevertheless exist, such as “uncertainty regarding malignant risk → illness perception → ruminative thinking → anxiety,” “uncertainty regarding malignant risk → ruminative thinking → illness perception → anxiety,” or “uncertainty regarding malignant risk → anxiety → illness perception/ruminative thinking.” Based on self-regulation theory, the present study hypothesized a sequential association of “uncertainty regarding malignant risk → illness perception → ruminative thinking → anxiety” and tested this hypothesized pathway to address the existing research gap.

## 1 Data and methods

### 1.1 Sample size calculation

The sample size for this study was calculated based on a power analysis for indirect effects in a serial mediation model. The two-sided significance level (α) was set at 0.05 and the statistical power (1 − β) was set at 0.80. Based on previous studies on psychological factors associated with pulmonary nodules (9, 10), the standardized effect sizes for each pathway in the model were conservatively assumed to be small to moderate (standardized path coefficients: a = 0.25, b1 = 0.25, and b2 = 0.25).

The serial mediation model included one independent variable and two serial mediators. In addition, 11 potential confounding factors—sex, age, educational level, marital status, monthly family income, smoking history, drinking history, number of nodules, nodule location, nodule density, and family history of lung cancer—were included as covariates in the analysis. A Monte Carlo simulation was performed using RStudio with 5,000 simulation iterations. The simulation was based on standardized regression coefficients, assuming a standardized block covariance matrix for the covariance structure of the variables. Eleven covariates were included in each regression equation. The serial indirect pathway MUIS → BIPQ → RRS → GAD-7 was specified as the target effect for power estimation, with simulation parameters determined based on effect sizes reported in previous studies (9, 10). The Monte Carlo simulation indicated that a minimum effective sample size of 230 participants was required to detect a small-to-moderate, serial, indirect effect. This estimate was based on the smallest indirect effect observed in the model. Allowing for a 20% rate of invalid questionnaires and attrition, the final required sample size was determined to be ≥288 participants. A total of 308 patients were ultimately included in the study, exceeding the minimum required sample size and satisfying the statistical power requirements for serial mediation analysis.

### 1.2 Participants

This study was approved by the Medical Ethics Committee of the Xinjiang Production and Construction Corps Hospital (approval number: 202207501). A total of 308 patients with pulmonary nodules who were admitted to our hospital between January 2023 and January 2026 were selected as participants. The inclusion criteria were ad follows: ① met the clinical diagnostic criteria for pulmonary nodules (11) and were diagnosed with pulmonary nodules by chest CT (nodule diameter ≤3 cm); ② age ≥18 years and ≤80 years; ③ basic reading and writing skills and ability to complete the questionnaire independently; and ④ complete clinical data. The exclusion criteria were as follows: ① previous history of lung cancer or other malignant tumors; ② complicated with serious organic diseases such as heart, liver, and kidney; ③ history of mental illness or use of antipsychotic drugs; ④ those with cognitive dysfunction or communication disorder; ⑤ Pregnant or lactating women; ⑥ had received surgical treatment of pulmonary nodules or biopsy to confirm the pathological diagnosis. All participants completed the questionnaire within one month after pulmonary nodules were detected on chest CT. At the time of questionnaire completion, the nature of the nodules remained undetermined, and none of the patients had received a definitive pathological diagnosis of the nodules. The patients had not yet received the results of the malignancy risk assessment and were awaiting either biopsy or follow-up evaluation.

### 1.3 Research tools

#### 1.3.1 General data collection

The general data statistics table was designed by the researchers, and the content validity was tested by three respiratory and psychological experts with associate senior or above titles. The content validity index (CVI) was 0.92 for the scale. Genera; patient data were collected from the hospital medical record system, including gender, age, education level, marital status, monthly household income, smoking history, drinking history, nodule number, nodule location, nodule density. and family history of lung cancer.

#### 1.3.2 Uncertainty regarding malignant risk scale

Based on items related to malignancy risk in the Mishel Uncertainty in Illness Scale (MUIS) (12), appropriate modifications were made to account for the characteristics of patients with pulmonary nodules. The revision process was as follows: Using the original item pool of the scale, the research team selected items closely related to uncertainty regarding malignancy risk based on the clinical characteristics of patients with pulmonary nodules and findings from previous interviews and adapted the wording accordingly. The final revised scale comprised 20 items across four dimensions: unpredictability, lack of information, complexity, and ambiguity. Of these, 12 items were retained in their original form, eight were modified, and 13 were deleted.

The revision process followed the established procedures for cross-cultural adaptation. First, two researchers in nursing and clinical psychology independently translated the scale from English to Chinese. Following discussions within the research team, an initial Chinese version was developed. A translator proficient in medical English independently back-translated the Chinese version into English. The back-translated version was compared with the original scale to identify semantic discrepancies and ensure that the core meaning of each item was consistent with that of the original scale.

Cognitive interviews were subsequently conducted with 15 patients with pulmonary nodules undergoing outpatient follow-up to assess the comprehensibility, relevance, and appropriateness of the items. Based on patient feedback, the wording of the three items was refined to minimize potential misunderstandings arising from technical terminology. Subsequently, 32 patients who met the inclusion criteria participated in a pretest to assess the acceptability and preliminary measurement properties of the revised version. The mean completion time was approximately 9 minutes, the valid response rate was 96.9%, and the preliminary Cronbach’s α was 0.886.

Following the revision, five senior experts, including three chief physicians in respiratory medicine and two associate physicians in clinical psychology, were invited to evaluate content validity. The item-level content validity index (I-CVI) ranged from 0.80 to 1.00, and the scale-level content validity index (S-CVI/Ave) was 0.91.

Exploratory factor analysis was conducted using data from 100 patients recruited before the formal study. These 100 patients were not included in the final sample of 308 patients. The Kaiser–Meyer–Olkin (KMO) value was 0.872, and Bartlett’s test of sphericity was significant (χ² = 1286.34, P< 0.001), indicating that the data were suitable for factor analysis. Principal component analysis identified four common factors, with item factor loadings ranging from 0.512–0.794. The four factors explained 62.18% of the cumulative variance, which is consistent with the four-dimensional structure of the original scale and indicates good structural validity.

The revised scale uses a 5-point Likert response format ranging from 1 (“strongly disagree”) to 5 (“strongly agree”), yielding total scores ranging from 20 to 100. Higher scores indicate greater uncertainty in the risk of malignancy. In the present study, the revised scale demonstrated an acceptable internal consistency (Cronbach’s α = 0.892).

#### 1.3.3 Brief illness perception questionnaire

The Brief Illness Perception Questionnaire (BIPQ) (13) was used for evaluation, which included eight dimensions: consequences, timeline, personal control, treatment control, disease identification, disease concern, disease understanding, and emotional representation, and an open etiological question. The first eight dimensions are graded from 0 to 10, in which the dimensions of personal control, treatment control, and disease understanding are reverse scores, the scores of the above three dimensions are counted into the total score after reverse conversion, and the rest of the dimensions are counted according to the original scores. The total score ranged from 0 to 80. A higher score indicates a more negative perception of the disease, indicating a greater degree of negative illness perception. The internal consistency of this scale in this study was acceptable (Cronbach’s α=0.831).

#### 1.3.4 Ruminative responses scale

The Chinese version of the Ruminating Thinking Scale (RRS) (14) was used for evaluation, which included three dimensions: symptom rumination, forced thinking, and introspection, with 22 items in total. Likert’s 4-grade scoring system was used, with 1–4 points from “never” to “always”, with the total score ranging from 22 to 88 points. The higher the score, the higher the ruminative thinking level of the patient. The internal consistency of this scale in this study was acceptable (Cronbach’s α=0.904).

#### 1.3.5 Generalized anxiety disorder 7-item scale

The Chinese version of the Generalized Anxiety Disorder 7-item scale (GAD-7) (15) was used to evaluate the scale, which consisted of seven items. Using Likert’s 4-level scoring method, 0–3 points were scored from “not at all” to “almost every day,” with the total score ranging from 0 to 21 points. Scores of 0–4 indicate no anxiety, 5–9 indicate mild anxiety, 10–14 indicate moderate anxiety, and 15–21 indicater severe anxiety. The internal consistency of this scale in this study was acceptable (Cronbach’s α=0.885).

### 1.4 Statistical methods

Data were analyzed using SPSS version 26.0. The normality of the continuous data was assessed using the Shapiro–Wilk test. Normally distributed data are expressed as mean ± standard deviation (SD). Comparisons between two groups were performed using the independent-samples t-test, while comparisons among multiple groups were performed using one-way analysis of variance (ANOVA), followed by Bonferroni correction for pairwise comparisons. Categorical data are expressed as n (%) and were compared using the χ² test.

According to the Shapiro–Wilk test, the MUIS and BIPQ scores met the assumption of normality (W = 0.992 and 0.992, respectively; P = 0.096 and 0.096, respectively), whereas the RRS and GAD-7 scores did not. However, given the relatively large sample size (n = 308) and the sensitivity of the Shapiro–Wilk test to minor deviations from normality, the distributions were further evaluated using histograms, skewness, and kurtosis coefficients. The absolute skewness values for all scales were<1, and the absolute kurtosis values were<2, indicating that the scale scores were approximately normally distributed. Moreover, total scale scores are generally treated as continuous variables in psychometric analysis. Therefore, Pearson’s correlation analysis was used to examine the correlations among uncertainty regarding malignancy risk, illness perception, ruminative thinking, and anxiety.

Serial mediation analysis was performed using Model 6 of the PROCESS macro. Eleven variables—age, sex, educational level, marital status, monthly family income, smoking history, drinking history, number of nodules, nodule location, nodule density, and family history of lung cancer—were included as covariates. Indirect effects were tested using bias-corrected bootstrap confidence intervals based on 5,000 resampling. An indirect effect was considered statistically significant if the 95% confidence interval (CI) did not include zero. A two-sided P< 0.05 was considered to be statistically significant.

## 2 Results

### 2.1 General information of the participants

A total of 308 patients with pulmonary nodules were included in this study, comprising 161 men (52.27%) and 147 women (47.73%). Of these, 222 patients were aged<60 years (72.08%), and 86 were aged ≥60 years (27.92%). Regarding educational level, 81 patients had a junior high school education or below (26.30%), 98 had a senior high school/technical secondary school education (31.82%), and 129 had a junior college education or above (41.88%). Regarding marital status, 264 patients were married (85.71%), and 44 were unmarried, divorced, or widowed (14.29%). The monthly household income was<5,000 yuan for 135 patients (43.83%), 5,000–10,000 yuan for 114 patients (37.01%), and >10,000 yuan for 59 patients (19.16%). A total of 101 patients had a history of smoking (32.97%), and 105 had a history of alcohol consumption (34.09%).

Regarding nodule characteristics, 189 patients had a single nodule (61.36%), and 119 had multiple nodules (38.64%). Nodules were located in the left lung in 136 patients (44.16%) and in the right lung in 172 patients (55.84%). Regarding nodule density, 92 patients had pure ground-glass nodules (29.87%), 75 had mixed ground-glass nodules (24.35%), and 141 had solid nodules (45.78%). A total of 47 patients had a family history of lung cancer (15.26%).

### 2.2 Scores of each scale

The MUIS scores of the 308 patients with pulmonary nodules ranged from 24 to 86, with an average of (56.10 ± 12.21). BIPQ scores 3-76, with an average of (43.26 ± 10.67), RRS scores 22-72, with an average of (44.66 ± 10.60), and GAD-7 scores 1-16, with an average of (8.84 ± 2.62) (**Table 1**).

**Table 1:** Scores of each scale ( $\bar{x}\pm s$).

Project	Score (points)
MUIS	56.10 ± 12.21
BIPQ	43.26 ± 10.67
RRS	44.66 ± 10.60
GAD-7	8.84 ± 2.62

### 2.3 Comparison of GAD-7 scores of patients with different demographic and disease characteristics

There was no significant difference in the GAD-7 scores of patients with different demographic and disease characteristics (*P*>0.05) (**Table 2**).

**Table 2:** Comparison of GAD-7 scores of patients with different demographic and disease characteristics ( $\bar{x}\pm s$).

Characteristic	Group	Number of cases	GAD-7 scale score	*t*/*F*	*P*
Gender	Male	161	8.86 ± 2.71	0.091	0.928
	Female	147	8.83 ± 2.53		
Age	<60 years old	222	9.00 ± 2.63	1.731	0.084
	≥60 years old	86	8.43 ± 2.57		
Degree of education	Junior high school and below	81	9.20 ± 2.69	1.617	0.200
	High school/technical secondary school	98	8.94 ± 2.61		
	College or above	129	8.55 ± 2.57		
Marital status	Married	264	8.86 ± 2.61	0.257	0.797
	Unmarried/divorced/widowed	44	8.75 ± 2.71		
monthly household income	<5000 yuan	135	8.60 ± 2.62	1.110	0.331
	5000~10000 yuan	114	8.98 ± 2.57		
	>10000 yuan	59	9.14 ± 2.73		
Smoking history	Yes	101	8.66 ± 2.68	0.801	0.424
	No	207	8.92 ± 2.60		
Drinking history	Yes	105	8.76 ± 2.83	0.627	0.531
	No	203	8.95 ± 2.31		
Nodule number	solitary nodule	189	8.81 ± 2.50	0.292	0.771
	multiple nodules	119	8.90 ± 2.81		
Nodular position	Left lung	136	8.95 ± 2.68	0.621	0.535
	Right lung	172	8.76 ± 2.57		
Nodular density	Pure ground glass nodule	92	8.72 ± 2.96	0.195	0.823
	Mixed ground glass nodule	75	8.83 ± 2.54		
	Solid nodule	141	8.94 ± 2.44		
Family history of lung cancer	Yes	47	8.57 ± 2.53	0.766	0.444
	No	261	8.89 ± 2.64		

### 2.4 Correlation analysis between variables

Pearson correlation analysis showed that the MUIS score was positively correlated with the BIPQ, RRS, and GAD-7 scores (*r* = 0.687, 0.577, and 0.745, respectively; *P* < 0.05). The BIPQ score was positively correlated with the RRS and GAD-7 scores (*r* = 0.577, 0.601, *P* < 0.05). The RRS score was positively correlated with the GAD-7 score (*r* = 0.574, *P* < 0.05) (**Table 3**, **Figure 1**).

**Table 3:** Correlation analysis between variables.

Variable	MUIS	BIPQ	RRS	GAD-7
MUIS	1	0.687^*^	0.577^*^	0.745^*^
BIPQ	0.687^*^	1	0.577^*^	0.601^*^
RRS	0.577^*^	0.577^*^	1	0.574^*^
GAD-7	0.745^*^	0.601^*^	0.574^*^	1

Pearson correlation analysis, ^*^P < 0.05.

**Figure 1:**
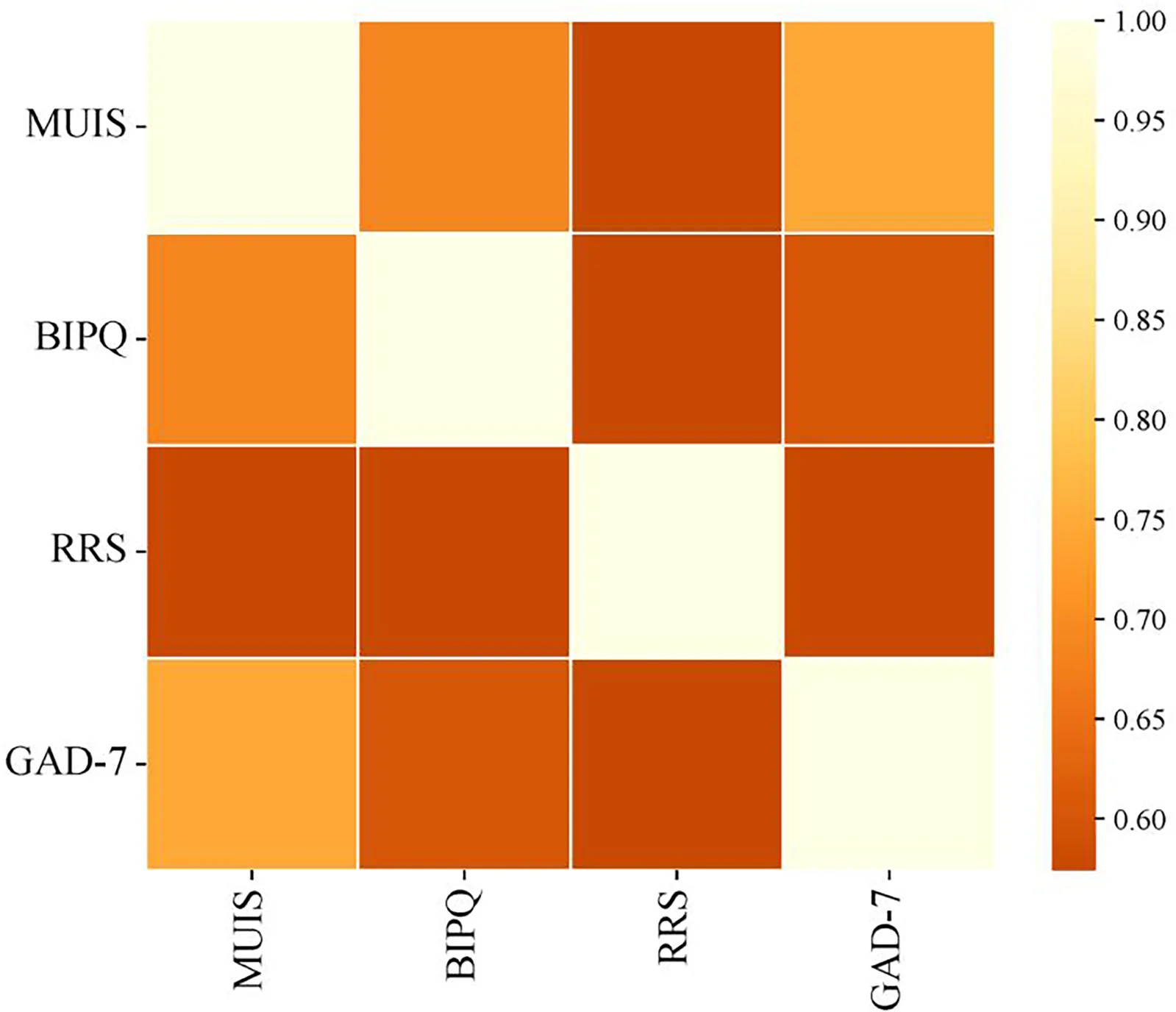
Correlation analysis between variables.

### 2.5 The serial indirect effect of illness perception and ruminative thinking between uncertainty regarding malignant risk and anxiety after detection of pulmonary nodules

Taking MUIS as the independent variable (X), GAD-7 as the dependent variable (Y), and BIPQ and RRS as the intermediate variables (M1, M2), a mediation effect model was constructed. The model shows that MUIS can positively predict GAD-7, BIPQ and RRS, BIPQ can positively predict GAD-7 and RRS, and RRS can positively predict GAD-7 (**Table 4**).

**Table 4:** The serial indirect effect of illness perception and ruminative thinking between uncertainty regarding malignant risk and anxiety after detection of pulmonary nodules.

Outcome variable	Predictor variable	Model coefficient						Model fitting indicators
		Non-standardized regression coefficient *B*	*SE*	Standardized *β*	*t*	*P*	95% *CI*	
GAD-7	MUIS	0.121	0.011	0.562	10.626	<0.001	0.098~0.143	*R*^2^ = 0.612 *F* = 33.003 *P* < 0.001
	BIPQ	0.027	0.013	0.112	2.103	0.036	0.002~0.053	*R*^2^ = 0.416 *F* = 16.083 *P* < 0.001
	RRS	0.047	0.012	0.188	3.958	<0.001	0.023~0.070	*R*^2^ = 0.694 *F* = 22.868 *P* < 0.001
RRS	MUIS	0.296	0.054	0.341	5.533	<0.001	0.191~0.402	*R*^2^ = 0.612 *F* = 33.003 *P* < 0.001
	BIPQ	0.347	0.062	0.349	5.638	<0.001	0.226~0.468	*R*^2^ = 0.416 *F* = 16.083 *P* < 0.001
BIPQ	MUIS	0.593	0.037	0.679	16.033	<0.001	0.520~0.666	*R*^2^ = 0.694 *F* = 22.868 *P* < 0.001

All the intermediary paths have been adjusted for age, gender, educational level, marital status, income, smoking history, drinking history, number of nodules, location of nodules, nodular density, and family history of lung cancer.

### 2.6 Testing the serial indirect effect of illness perception and ruminative thinking between the uncertainty regarding malignant risk and anxiety after the detection of pulmonary nodules

MUIS had a significant direct effect on GAD-7, accounting for 75.63% of the total effect. The indirect effects consisted of three paths: ①MUIS→RRS→GAD-7, with an indirect effect value of0.014, accounting for 8.75% of the total effect; ②MUIS→BIPQ→GAD-7, with an indirect effect value of 0.016, accounting for 10.00% of the total effect; and ③MUIS→BIPQ→RRS→GAD-7, with an indirect effect value of 0.009, accounting for 5.62% of the total effect. The above-mentioned effects were non-standardized. The effect proportion indicates the breakdown ratio of each direct and indirect effect to the total effect (**Table 5**, **Figure 2**).

**Table 5:** Testing the serial indirect effect of illness perception and ruminative thinking between the uncertainty regarding malignant risk and anxiety after the detection of pulmonary nodules.

Effect	Effect path	Effect value	SE	95% *CI*	Effect proportion (%)
Total effect	MUIS→GAD-7	0.160	0.008	0.144~0.176	100
Direct effect	MUIS→GAD-7	0.121	0.011	0.099~0.143	75.63
Indirect effect	MUIS→RRS→GAD-7	0.014	0.006	0.005~0.027	8.75
	MUIS→BIPQ→GAD-7	0.016	0.008	0.001~0.031	10.00
	MUIS→BIPQ→RRS→GAD-7	0.009	0.003	0.004~0.017	5.62

The indirect effects in the table are all unstandardized indirect effects, and the 95% confidence intervals are estimated using the bias-corrected Bootstrap method (with 5000 replications). All mediating paths have been corrected for age, gender, education level, marital status, income, smoking history, drinking history, number of nodules, location of nodules, nodular density, and family history of lung cancer.

**Figure 2:**
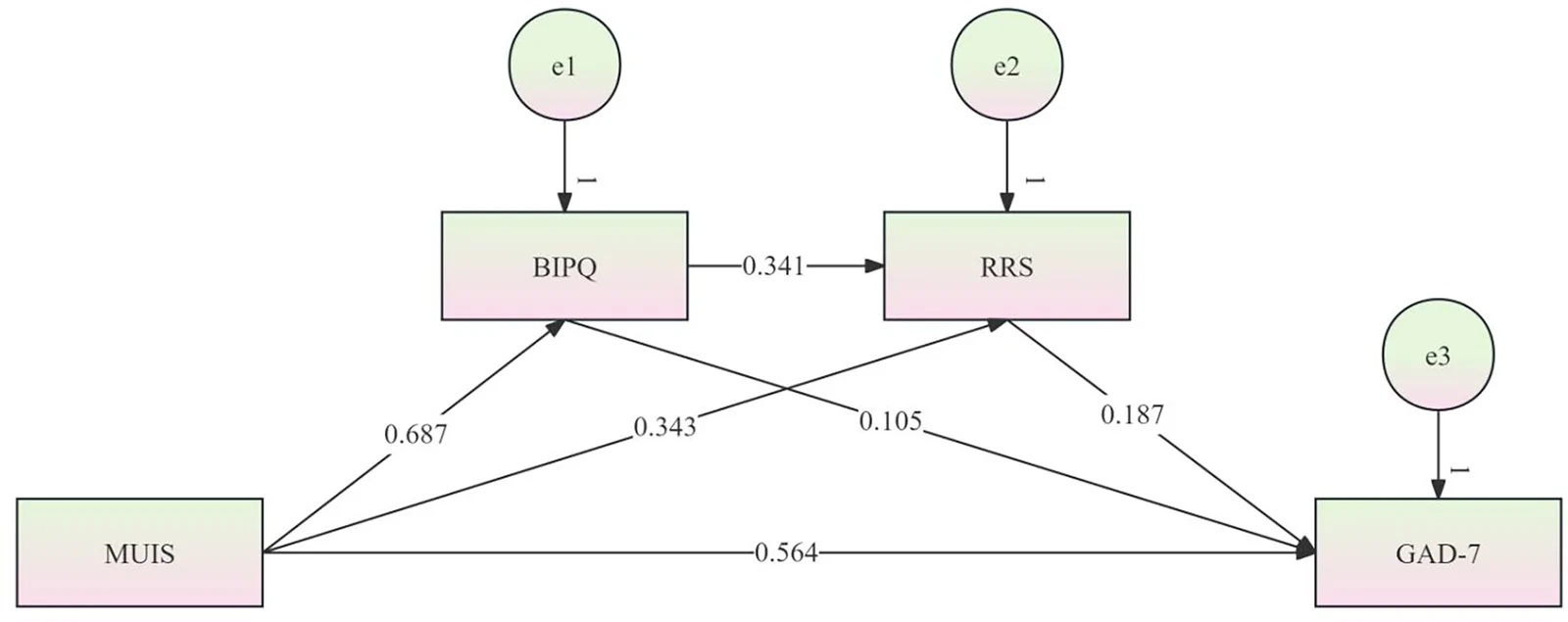
Test of the serial indirect effect of illness perception and ruminative thinking between the uncertainty regarding malignant risk after lung nodule detection and anxiety.

## 3 Discussion

### 3.1 Disease background

The imaging manifestations of pulmonary nodules are focal, quasi-circular, with a maximum diameter of ≤3 cm, mostly surrounded by air-containing lung parenchyma, without atelectasis, pleural effusion, or hilar lymphadenopathy (16). At present, the pathogenesis of the disease is not clear, and it is thought to be related to genetic susceptibility, epigenetic regulation, immune microenvironment remodeling, and environmental exposure (17). Approximately 95% of pulmonary nodules are benign lesions; however, some pulmonary nodules have the potential for malignant transformation. Approximately 20%-40% of non-solid pulmonary nodules continue to increase or show increased CT within 4 years, which may progress to invasive adenocarcinoma (18). Although most incidentally detected pulmonary nodules are ultimately confirmed to be benign, their detection and the subsequent process of ruling out malignancy may cause patients to experience negative emotions such as anxiety and depression, resulting in psychological distress. In response to this clinical concern, the present study constructed a serial mediation model to explore the sequential cognitive pathway involving illness perception and ruminative thinking in the association between uncertainty about malignancy risk and anxiety. The findings are expected to provide a basis for developing psychological interventions for patients with pulmonary nodules.

### 3.2 Status quo of uncertainty regarding malignant risk, illness perception, ruminative thinking and anxiety in patients with pulmonary nodules after detection

Studies have shown that patients with pulmonary nodules generally experience different degrees of anxiety (19). The MUIS score of the 308 patients with pulmonary nodules included in this study was 56.10, which is in the middle and high level, indicating that most patients have obvious experience of uncertainty regarding malignant risk after pulmonary nodules are detected, which is similar to Guo et al. (9). The high uncertainty in patients with pulmonary nodules is mainly due to the fuzziness of the imaging characteristics of pulmonary nodules, malignant risk assessment, and unpredictability of disease outcomes during long-term follow-up. Previous studies have suggested that when individuals are confronted with unpredictable threats, the amygdala may become hyperactivated, whereas the executive control functions of the prefrontal cortex may be impaired. These changes may hinder the effective integration of information and the formation of clear cognitive judgments, thereby contributing to heightened uncertainty (20). However, the present study did not assess relevant neurobiological indicators; therefore, this potential mechanism remains speculative and is based solely on evidence from existing literature. The average BIPQ score of patients with pulmonary nodules included in this study was 43.26, suggesting that patients with pulmonary nodules generally have a negative illness perception. Patients may overestimate the risk of malignancy and potential consequences of pulmonary nodules while underestimating their ability to manage the condition. Negative illness perceptions are closely related to the quality of information that patients receive. Many patients obtain fragmented or exaggerated information about lung cancer from non-professional sources, particularly the Internet, which may contribute to the misconception that pulmonary nodules are equivalent to lung cancer. Such misperceptions may, in turn, influence patients’ emotional responses and coping behaviors. Further analysis of ruminative thinking levels showed that the average RRS score of patients in this study was (44.66 ± 10.60). Ruminative thinking is a maladaptive cognitive style that is more easily activated in the face of uncertain disease threats, trapping patients in a negative thinking cycle. Neuroimaging research shows that ruminative thinking is related to the overactivation of default pattern network and the low function of dorsolateral prefrontal cortex, and it is also related to the difficulty for individuals to shift their attention from negative information (21). This study also found that the average GAD-7score in 308 patients was 8.84, which was at the level of mild anxiety but close to the critical value of moderate anxiety, suggesting that anxiety problems were common in patients with pulmonary nodules. However, the scores of GAD-7 in patients with different demographic and disease characteristics did not show statistical significance, which was different from previous studies (22). The analysis indicates that the early detection stage of pulmonary nodules may lead to uncertainty regarding the risk of malignancy, which is an important factor contributing to patients’ anxiety. The association between this factor and anxiety is stronger than that of age, gender, and the characteristics of the nodules.

### 3.3 Correlation among uncertainty regarding malignant risk, illness perception, ruminative thinking and anxiety

The association between uncertainty regarding malignancy risk and negative emotions has been demonstrated in various populations, including individuals with positive cancer screening results and those with chronic diseases (23, 24). The present study showed that uncertainty regarding the malignancy risk of pulmonary nodules was positively correlated with illness perception, ruminative thinking, and anxiety levels. Illness perception was also significantly positively correlated with ruminative thinking and anxiety, whereas ruminative thinking was significantly positively correlated with anxiety.

These positive correlations may reflect the dysregulation of cognitive-emotional processes when individuals are confronted with ambiguous health threats. As discussed above, previous studies have suggested that uncertainty regarding malignancy risk may be associated with the sustained activation of emotion-related brain regions, such as the amygdala and insula, as well as reduced executive control by the dorsolateral prefrontal cortex. Individuals may also exhibit selective attentional biases and enhanced processing of threat-related information (20). Unlike the definitive diagnosis of malignancy, the uncertain malignant potential of pulmonary nodules creates a persistent threat that cannot be immediately resolved through targeted medical intervention. This prolonged state of psychological uncertainty may maintain the threat perception system at a heightened level of arousal, thereby amplifying patients’ subjective perceptions of disease-related threats.

Illness perception refers to an individual’s cognitive representation of disease-related information. A high level of uncertainty regarding malignancy risk may interfere with an individual’s accurate assessment of the disease course, prognosis, and controllability, contributing to a negative illness perception characterized by a high perceived threat and low perceived control. Specifically, patients may overestimate the likelihood that pulmonary nodules are malignant, attribute nonspecific somatic symptoms, such as cough and chest tightness, to malignant disease, and underestimate their own ability and that of medical interventions to manage the condition. Such biased perceptions may be associated with increased anxiety.

Ruminative thinking may represent a maladaptive cognitive strategy that arises when individuals are unable to resolve uncertainty regarding disease-related threats. Patients may repeatedly review their examination results, anticipate adverse outcomes, and excessively worry about the future. These processes may enhance amygdala responses, consume prefrontal cognitive resources, and impair emotional regulation (25). Simultaneously, negative illness perceptions and ruminative thinking may interact with and reinforce each other, potentially creating a cycle of “uncertainty regarding malignancy risk → negative illness perception → ruminative thinking → anxiety,” which may be associated with greater anxiety severity.

### 3.4 The serial indirect effect of illness perception and ruminative thinking between uncertainty regarding malignant risk and anxiety after detection of pulmonary nodules

The results of this study showed that illness perception and ruminative thinking had both independent and serial indirect effects on the association between uncertainty regarding the risk of malignancy and anxiety in patients with pulmonary nodules, consistent with the hypothesized model. These findings suggest that the association between uncertainty regarding malignancy risk and anxiety may involve several pathways. However, given the cross-sectional design of this study, these indirect effects represent associations that are consistent with the hypothesized model and cannot establish temporal or causal relationships between the variables.

From the perspective of the dynamic process of cognitive-emotional regulation, uncertainty regarding malignancy risk, as an ambiguous threat that cannot be immediately resolved, may interfere with individuals’ cognitive appraisal processes and make it difficult for them to establish a clear and stable cognitive representation of their condition. Therefore, it may be associated with anxiety through three different pathways. Nevertheless, the proposed direction of these associations is based on self-regulation theory, and their temporal ordering cannot be established using cross-sectional data alone.

Regarding the direct pathway, previous studies have suggested that uncertainty regarding malignancy risk, as a chronic psychological stressor, may result in sustained activation of the hypothalamic–pituitary–adrenal axis and sympathetic nervous system, leading to increased cortisol and catecholamine levels, potentially contributing to anxiety-related physiological arousal and psychological experiences (26). However, the present study did not assess relevant biological indicators; therefore, this mechanism should be considered only as a potential explanation based on existing literature.

For the indirect pathway MUIS → RRS → GAD-7, the perceived loss of control associated with uncertainty regarding the risk of malignancy may prompt individuals to engage in repetitive thinking in an attempt to reduce uncertainty. However, because the malignancy risk of pulmonary nodules is inherently uncertain, such repetitive thinking may fail to provide definitive answers, potentially reinforcing rumination and contributing to persistent and severe anxiety.

For the indirect pathway MUIS → BIPQ → GAD-7, uncertainty regarding malignancy risk may interfere with the integration and appraisal of disease-related information, leading individuals to selectively process negative information, overestimate disease severity and the likelihood of malignancy, and underestimate their own ability and that of medical interventions to control their condition. These cognitive processes may contribute to negative illness perceptions, which may, in turn, be associated with greater anxiety.

The serial indirect pathway further suggests that negative illness perceptions may narrow individuals’ attentional focus toward disease-related negative cues and interfere with engagement in adaptive coping behaviors, thereby promoting and sustaining rumination. Rumination may, in turn, repeatedly activate and reinforce negative disease-related thoughts and memories, potentially exacerbating negative illness perceptions and creating a self-reinforcing cycle associated with persistent anxiety. However, this proposed dynamic process remains a theoretical interpretation of the observed associations and requires confirmation through longitudinal studies.

### 3.5 Clinical significance and limitations

Mental health problems among patients with pulmonary nodules have become an important public health concern, affecting patients’ quality of life and potentially contributing to excessive healthcare utilization and unnecessary consumption of medical resources. In current clinical practice, greater emphasis is often placed on physical health than on psychological well-being, resulting in insufficient attention to the psychological needs of patients with pulmonary nodules and a lack of targeted psychological interventions. The findings of this study provide a theoretical basis for developing appropriate psychological interventions for this patient group.

These findings suggest that clinicians should pay particular attention to uncertainty regarding the risk of malignancy among patients with pulmonary nodules and the associated psychological distress. Following the detection of pulmonary nodules, patients should be promptly provided with accurate and understandable information, including clear explanations of the nature of the nodules, the estimated risk of malignancy, and the follow-up plan to help reduce uncertainty. Illness perception and ruminative thinking may also be important targets for psychological interventions. Cognitive behavioral therapy may help patients identify and modify maladaptive cognitions, while mindfulness training, attentional redirection, and other approaches may help reduce ruminative thinking and interrupt maladaptive cognitive-emotional cycles.

This study has several strengths. First, the research question has direct clinical relevance, particularly given the increasing recognition of psychological distress among patients with indeterminate pulmonary nodules in the context of widespread computed tomography (CT) screening. Second, rather than focusing solely on pairwise correlations, this study proposed a theoretically informed serial indirect effect model, providing a more comprehensive framework for understanding the associations among the study variables. Third, the sample size was determined based on a power analysis for indirect effects and met the requirements for testing the proposed model. Fourth, illness perception, ruminative thinking, and anxiety were assessed using established instruments that demonstrated acceptable internal consistency in our sample. Finally, the study received institutional ethics approval, and written informed consent was obtained from all the participants.

However, this study has several limitations. First, the cross-sectional design meant that all variables were measured at approximately the same time, precluding the determination of their temporal sequence and causal relationships. Therefore, the indirect effects identified in this study should be interpreted only as correlational indirect effects consistent with the hypothesized model and not as evidence of causal mediation. In addition, although a sequential relationship of “illness perception → ruminative thinking” was hypothesized based on self-regulation theory, other plausible sequences may exist, such as “uncertainty regarding malignancy risk → ruminative thinking → illness perception → anxiety.” Cross-sectional data cannot distinguish between these alternative pathways. Therefore, future longitudinal studies are needed to further examine the temporal and dynamic relationships among uncertainty regarding malignancy risk, illness perception, ruminative thinking, and anxiety in patients with pulmonary nodules. Second, this was a single-center study conducted at one hospital, which may limit the representativeness of the sample and generalizability of the findings. Future multicenter studies with larger and more diverse samples are required. Third, data were collected using self-report instruments and may therefore be subject to recall and social desirability bias. Future studies should incorporate clinical interviews and physiological measures to provide a more comprehensive assessment of patients’ psychological status. Fourth, the MUIS was adapted for use in the present study, and further evidence regarding the psychometric properties of the revised scale in patients with pulmonary nodules, including its content validity, construct validity, and factor structure, is needed. Future studies should comprehensively psychometrically validate the revised instrument. Finally, no neurobiological indicators were assessed in this study. Therefore, the potential mechanisms discussed above, including alterations in brain region activity and neuroendocrine responses, are based on mechanisms proposed in previous studies and cannot be confirmed by the present data.

### 3.6 Summary

Following the detection of pulmonary nodules, uncertainty regarding malignancy risk was positively associated with anxiety, and this association was partly explained by the serial indirect effects of illness perception and ruminative thinking, consistent with the hypothesized model. In clinical practice, greater attention should be paid to the cognitive and psychological status of patients with pulmonary nodules. Comprehensive interventions aimed at reducing illness uncertainty and modifying negative illness perceptions may help improve the psychological well-being of patients with IBD.

## Data Availability

The original contributions presented in the study are included in the article/supplementary material. Further inquiries can be directed to the corresponding author.
